# Anion-Induced Structural Diversity and Optical Chromism in a Series of Cyano-Bridged Heterometallic 3*d*-4*f* Coordination Polymers

**DOI:** 10.3390/molecules28062871

**Published:** 2023-03-22

**Authors:** Flavia Artizzu, Luca Pilia, Angela Serpe, Dimitrije Mara, Maria Francesca Casula, Luciano Marchiò, Paola Deplano

**Affiliations:** 1 Dipartimento per lo Sviluppo Sostenibile e la Transizione Ecologica (DiSSTE), Università del Piemonte Orientale “A. Avogadro”, Piazza S. Eusebio 5, 13100 Vercelli, Italy; 2 Dipartimento di Ingegneria Meccanica Chimica e dei Materiali, Università di Cagliari, Via Marengo 2, 09123 Cagliari, Italy; 3 Dipartimento di Ingegneria Civile, Ambientale e Architettura, INSTM Research Unit, Università di Cagliari, Via Marengo 2, 09123 Cagliari, Italy; 4 Institute of General and Physical Chemistry, Studentski Trg 12/V, 11158 Belgrade, Serbia; 5 Dipartimento SCVSA, Università di Parma, Parco Area delle Scienze 17/a, 43124 Parma, Italy

**Keywords:** iron complexes, lanthanide, solvatochromism, coordination polymers, heterometallic assemblies

## Abstract

The self-assembly reaction of the neutral dicyano-bis(1,10-phenanthroline) iron(II) complex with lanthanide ions (Ln = Eu(III), Gd(III), Er(III)) provided two different classes of heterometallic cyano-bridged 3*d*-4*f* architectures depending on the nature of the counteranion, irrespective of the size of the 4*f* metal ion. Tetranuclear oligomers with a squared Fe_2_Ln_2_ core were isolated when using nitrate salts, whereas unusual 1D polymeric chains were obtained when resorting to triflate salts under the same synthetic conditions. It is shown that the different structural motifs have a remarkable impact on the thermal stability and the optical properties of the compounds, which display a notable optical ipsochromism of the parent Fe(II) complex upon coordination with the Ln ion. This effect is significantly more pronounced in the polymeric chain than in the Fe_2_Ln_2_ oligomer both in solution and in the solid state. Structural evidence suggests that this behavior is likely related to the geometry of the CN-Ln bridge. On the other hand, more extended π-stacking interactions in the oligomer give rise to a broad charge-transfer absorption (600–1500 nm), making this compound promising as NIR absorber. Density Functional Theory calculations and electrochemical studies demonstrate that the observed negative chromism originates from the stabilization of a mixed metal/cyanide character HOMO with respect to a phenanthroline-centered LUMO.

## 1. Introduction

Coordination polymers and metal–organic frameworks are receiving great attention because of their fascinating architectures and topologies that bestow excellent functionalities in catalysis, sensing, gas storage and chemical separation on these materials, while enhancing the chemical and thermal stability due to the rigidity of the extended (1D to 3D) molecular structure. Moreover, the incorporation of transition metal (3*d*) and lanthanide (4*f*) ions, particularly in heterometallic 3*d*-4*f* assemblies, can enrich such compounds with additional unique optical and magnetic properties, including tunable colored and white-light generation, near-infrared (NIR) emission, ferromagnetism and slow relaxation of the magnetization [1,2,3,4,5,6]. Supramolecular interactions, such as π-stacking and hydrogen bonding, can have a relevant direct impact on the physical properties of the materials, as well as indirect effects, for example, by favoring distance-dependent through-space phenomena, such as Förster’s energy transfer [7,8,9,10,11]. However, controlling the assembly of coordination polymers and extended molecular frameworks is challenging, particularly when lanthanide (Ln) ions are involved, since their coordination chemistry is essentially governed by entropic and steric factors rather than orbital directionality. Typically, for established synthetic conditions and organic linkers, changes in the structural motif are determined by the nature of the Ln ion, specifically related to the size, where the early terms of the Ln series up to the middle-term gadolinium (light lanthanides) can usually accommodate more binding donor atoms because of their larger radius [12,13]. Exceptions to this trend exist, where even close-sized Ln ions, such as erbium and ytterbium, template remarkably different architectures, underlining the difficult predictability of the resulting compound [14]. On the other hand, the role of the (non-linker) counteranion in determining the structural arrangement of polynuclear molecular compounds is relatively less explored, especially in regard to Ln ions [15,16,17]. In this work, we present a rare study, where the structure and morphology of the coordination network are dictated by the counteranion while no discrimination is established based on the size difference of heavy and light Ln ions. We selected the neutral [Fe(Phen)_2_CN_2_] (Phen = 1,10-phenanthroline) metalloligand to build heterometallic molecular architectures with Ln ions belonging to both the first and second half of the Ln series. This precursor molecular complex takes on a particular relevance since it has been recently demonstrated as an ideal candidate to produce a rigid backbone for the equatorial position of *f*-elements with large magnetic anisotropy, thus providing stabilization of the easy axis of magnetization of potential single-molecule magnets [4]. We have reinvestigated the synthesis, crystal structure and the properties of this popular metalloligand and we show that, under identical synthetic conditions, the reaction with the nitrate and triflate hydrated salts of Eu(III), Gd(III) and Er(III) affords neutral discrete tetranuclear assemblies and unusual 1D coordination polymers, respectively. It is shown that the different structural arrangement of these two classes of compounds, dictated by the different counteranions, has a significant impact on their chemical and physical properties, especially in regard to the remarkable ion-dependent optical chromism. In-depth structural, spectroscopic and Density Functional Theory studies highlight the main factors that govern the observed behavior.

## 2. Results and Discussion

### 2.1. Synthesis and Crystal Structure Description

**Scheme 1:**
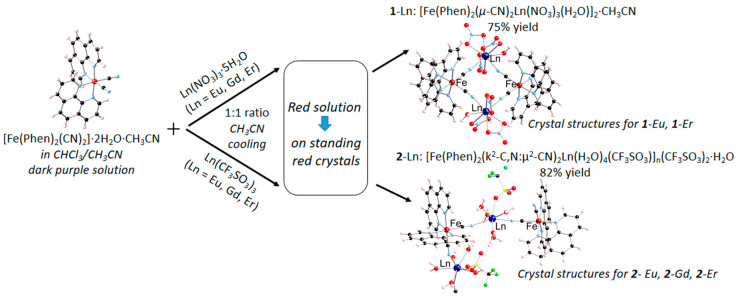
Synthetic pathways for the heterometallic compounds **1**-Ln and **2**-Ln.

The precursor dicyano-bis(1,10-phenanthroline) iron(II) complex was synthesized through a simplified procedure with respect to that originally reported by Schilt in 1960 [18] by purifying the crude product by slow recrystallization in acetonitrile. X-ray structural studies on single crystal yielded a product of formula [Fe(Phen)_2_(CN)_2_]·2H_2_O·CH_3_CN. The two cyanide groups are in the *cis* position and share hydrogen bonding interactions with two water molecules of hydration (Figure S1). The structure differs from the previous reports for the presence of one acetonitrile crystallization molecule [19,20]. Crystal structure and data for this compound are reported in Supplementary Information (Figure S1 and Table S1). As a result of the reaction of the above-described metalloligand with Ln salts, two classes of heterometallic Fe(II)/Ln(III) molecular architectures have been obtained, as summarized in Scheme 1. The heterobimetallic tetranuclear neutral complexes [Fe(phen)_2_(*µ*-CN)_2_Ln(NO_3_)_3_(H_2_O)]_2_·CH_3_CN (**1**-Ln; Ln = Eu, Gd, Er, Phen = 1,10-phenanthroline) are precipitated as red solids after slow solvent evaporation following the reaction of [Fe(Phen)_2_(CN)_2_] with Ln(NO_3_)_3_·nH_2_O (1:1 molar ratio) in CHCl_3_/CH_3_CN solvent mixture at room temperature. Instead, by applying the same synthetic conditions, the reaction of the triflate salt of the Ln ion, Ln(CF_3_SO_3_)_3_·nH_2_O (Ln = Eu, Gd, Er), with the same iron(II) precursor produces the heterometallic 1D coordination polymer [Fe(Phen)_2_(k^2^-C,N:μ^2^-CN)_2_Ln(H_2_O)_4_(CF_3_SO_3_)]_n_(CF_3_SO_3_)_2_·H_2_O (**2**-Ln). The composition and stoichiometry of the obtained compounds are confirmed by analytical and spectroscopic investigation (Section 3), whereas X-ray structural studies on single crystals have been performed for compounds **1**-Eu, **1**-Er, **2**-Eu, **2**-Gd, **2**-Er. A summary of data collection and structure refinement for **1**-Eu, **1**-Er and **2**-Eu, **2**-Gd, **2**-Er is reported in Table 1 and Table 2, respectively.

**Figure 1:**
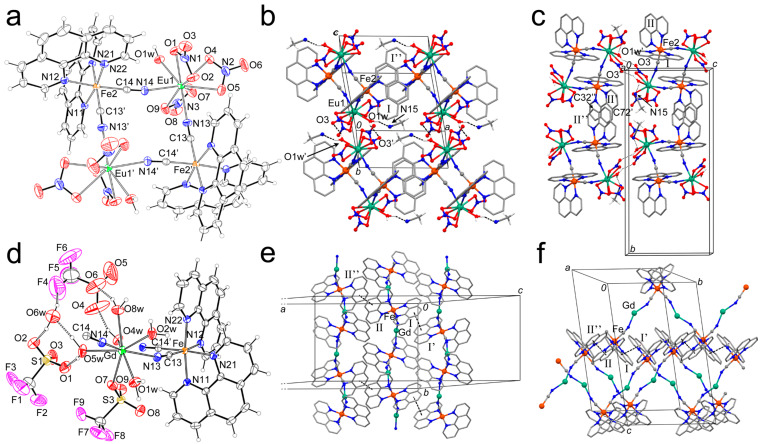
(**a**) Molecular drawing of **1**-Eu at the 30% ellipsoids probability level. The acetonitrile crystallization molecules have been removed for clarity. (**b**,**c**) Views of the crystal packing of **1**-Eu, where hydrogen bonds between water molecules, nitrate anions and acetonitrile molecules are reported as dashed bonds. The **I** phenanthroline moieties are regularly π-stacked with two out of three aromatic rings. The packing along *b* is determined by the interaction between the symmetry-related **II** phenanthroline groups. (**d**) Molecular drawing of **2**-Gd at the 30% ellipsoids probability level. Hydrogen bonds between water molecules and triflate anions are reported as dashed bonds. (**e**,**f**) Views of the crystal packing of **2**-Gd. The π-stacking between **I** and **II** phenanthroline rings is reported as dashed bonds. The triflate counteranions and the crystallization water molecules have been removed for clarity.

**Figure 2:**
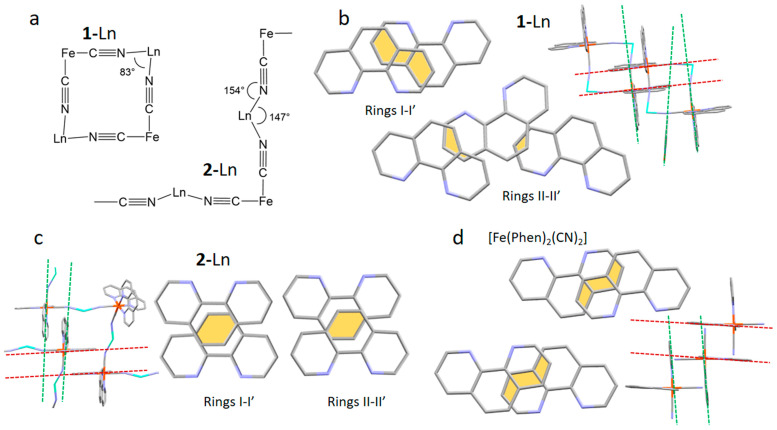
(**a**) Comparison of the tetranuclear and chain-like arrangement of compounds **1**-Ln and **2**-Ln and (**b**–**d**) schematic representation of the overlap (evidenced in yellow) of phenanthroline rings related by short contact interactions (π-stacking) in **1**-Ln, **2**-Ln and [Fe(Phen)_2_(CN)_2_], respectively. Dashed lines represent the phenanthroline rings’ planes.

The X-ray characterization reveals that compounds **1**-Eu and **1**-Er are isostructural, whereas **2**-Gd is isostructural with **2**-Eu and **2**-Er. No structural discrimination is observed based on the radii of light (Eu), heavy (Er) or mid-term (Gd) Ln ions. The structures of **1**-Eu and **2**-Gd, reported in Figure 1 and Figures S1–S4, are taken as representative and discussed here in detail. Powder-XRD patterns are reported in Figure S5. As can be seen, the most striking difference between the two classes of compounds is the discrete oligometallic architecture of **1**-Ln (Figure 1a) and the polymeric nature of **2**-Ln (Figure 1e) induced by the different arrangement of the bridging cyanide groups (Figure 2a), as discussed further.

The ORTEP diagram of **1**-Eu is depicted in Figure 1a and selected bond distances and angles of **1**-Eu and **1**-Er are reported in Table S2. The molecular assembly of **1**-Eu can be thought of as a square, where the opposite vertex is occupied by Eu and Fe atoms and with the sides that are represented by bridging cyanide anions. The octahedral coordination of Fe is achieved by means of two N,N-chelate phenanthroline ligands and by two C-bonded cyanide groups in the *cis* position. The Fe-N bond distances that are in the *trans* position to the CN^−^ units are significantly longer than the remaining Fe-N distances, in agreement with the *trans*-influence exerted by the cyanide. The europium ion is surrounded by three O,O-chelate nitrate anions, a water molecule and two N-bonded CN^−^ groups. The coordination geometry of the europium ion is difficult to describe due to the restraints imposed by the chelating nitrate anions (bite angle of ~50°) that severely distort the ideal geometry. A picture of the Eu environment, which can be defined as capped square anti-prismatic, with the square faces represented by O(2)-O(4)-O1w-N(14) and by O(5)-O(7)-O(9)-N(13) and with O(1) that is capping the former face, is reported in Figure S2. The Eu-donor atom bond distances are in the 2.429(3)–2.538(2) Å range.

The crystal packing of **1**-Eu shows that the molecules are arranged in layers that are parallel to the *ac* plane (Figure 1b). This 2D assembly is generated by two different types of supramolecular contacts. In particular, two hydrogen bonds between the O1w and the O(3)_nitrate_ atom (2.934(4) Å) determine the packing along the *c* axis, whereas the regular π-stack between one of the two phenanthroline units (**I**) determines the packing along the *a* axis. Two out of three aromatic rings of **I** are involved in the latter interaction, with the distance between the centroids of the overlapping rings of 3.463(4) Å. Furthermore, the 2D layers that are parallel to the *ac* plane interact with each other by means of the **II** phenanthroline moieties (Figure 1c). In this case, the π-stack is not regular since these aromatic ligands are slightly diverging (Figure 2b). The minimum distance between the **II** planes is 3.476(6) Å and it is determined by the C(32) and C(72)^#^ atoms (symmetry code # = ½ + x; ½ − y; z).

The coordination bond distances of **1**-Er are, on average, 0.04 Å shorter than in **1**-Eu, in accordance with the smaller ionic radii of the Er(III) ion with respect to Eu(III) (Table S1) [21].

The ORTEP drawing of **2**-Gd is reported in Figure 1d, whereas selected bond lengths and angles for **2**-Gd, **2**-Eu and **2**-Er are reported in Table S3. The structure of **2**-Gd presents some similarities with that of **1**-Eu, and these are related to the coordination environment of Fe and to the bridging behavior of the CN^−^ anions that are N-bonded to the lanthanide and C-bonded to the iron atoms, respectively. As for **1**-Eu, the Fe-N bond distances in *trans* to the CN^−^ groups are significantly longer than the remaining Fe-N distances. The presence of triflate anions in place of the nitrate ions found in **1**-Eu has important consequences on the coordination geometry of the Ln ion. In fact, since the triflate anions usually behave as O-monodentate, to reduce the steric hindrance around the metal, five water molecules are metal-bound and only one triflate ion is coordinated through one oxygen atom (O(7)). The remaining triflate groups are linked through hydrogen bonds (HB) to the coordinated and co-crystallized (O6w) water molecules. In particular, O5w behaves as an HB donor with respect to O6w and O(1), O4w as an HB donor with O(4), O8w acts as an HB donor with O(6) and O6w behaves as an HB donor with O(2) and F(4). Since the Gd coordination is achieved by means of monodentate ligands, the metal geometry is much more regular than in **1**-Eu and can be described as square anti-prismatic (Figure S3), with the square faces represented by O1w-N(14)-O5w-O(7) and by O2w-O3w-O4w-N(13) atoms. The Gd-donor atom bond distances are in the range 2.389(3)–2.479(3) Å, and the coordination bond distances of **2**-Ln are in the order **2**-Eu > **2**-Gd >**2**-Er in agreement with the ionic radii order r(Eu^3+^) > r(Gd^3+^) > r(Er^3+^) (Table S3) [22].

The two cyanide ions surrounding Gd(III) point approximately in opposite directions with the N-Gd-N angle of ~150°. This disposition of the CN^−^ groups is responsible for the polymeric nature of **2**-Ln, which can be described as a zigzag chain (Figure S4). On the other hand, in **1**-Eu, the N-Eu-N angle is ~83° with the consequence that the resulting structure is tetranuclear, Figure 2a. The molecular chains in **2**-Gd run parallel to the *c* axis and they interact with symmetry-related ones by means of π-stacks using both phenanthroline moieties (**I** and **II**), Figure 1e,f and Figure S4. Differently from **1**-Eu, this π-stack occurs only through the central aromatic ring, with the distance between the two ring centroids of 3.506(5) Å for the **I**-**I** stack and 3.545(9) Å for the **II**-**II** stack, respectively (Figure 2c).

From a literature survey, only the complex [Fe(Phen)_2_(*μ*^2^-CN)_2_YbCl_3_(H_2_O)]_2_ exhibits the cyano-bridged dinuclear arrangement found in **1**-Eu, and it is composed of Fe(Phen)_2_^2+^ units and Yb(III) ions occupying the vertex of a square [22]. A similar Ln_2_Fe_2_ squared topology is found in {Dy(CH_3_OH)_4_[Fe(*μ*^2^-CN)_2_(Phen)_2_]_2_}(CF_3_SO_3_)_3_·5CH_3_OH and its derivative{Dy(pyNO)_2_(CH_3_OH)_4_[Fe(*μ*^2^-CN)_2_(Phen)_2_]_2_}(CF_3_SO_3_)_3_·1.5CH_3_OH (pyNO = pyridine N-oxide) but the squares share a vertex corresponding to the dysprosium ions resulting in a 1D coordination polymer [4]. The only other report of a heterometallic Fe(II)/Ln(III) compound is {Yb(pyNO)_3_[Fe(*μ*-CN)_2_(Phen)_2_]_2_}(CF_3_SO_3_)_3_·2CH_3_CN, which has a similar stoichiometry with respect to the compounds reported in reference [4] but consists of a discrete trinuclear chain structure, where the Fe(II) precursor complex acts as a monodentate ligand, leaving one cyanide group free [5].

From a comparison of the bimetallic molecular structures reported in the literature with similar building blocks, i.e., N,N bidentate ligands (phenanthroline or bipyridine), Fe(III) or Fe(II), Ln(III) and CN^−^, it appears that the iron precursor is incorporated *as is* in the resulting molecular arrangement, irrespective of the iron oxidation state (+2 or +3), where tetranuclear squared topologies seem to be predominant when the iron precursor is coordinated to a blocking ligand such as Phen, as in **1**-Ln [23,24,25] whereas coordination polymers are only obtained when using [Fe(CN)_6_]^3−^ as precursor [26,27,28,29]. In this respect, the 1D polymeric structure of **2**-Ln represents a unique case, likely induced by the steric hindrance of the triflate anion.

### 2.2. Vibrational Spectroscopy

**Figure 3:**
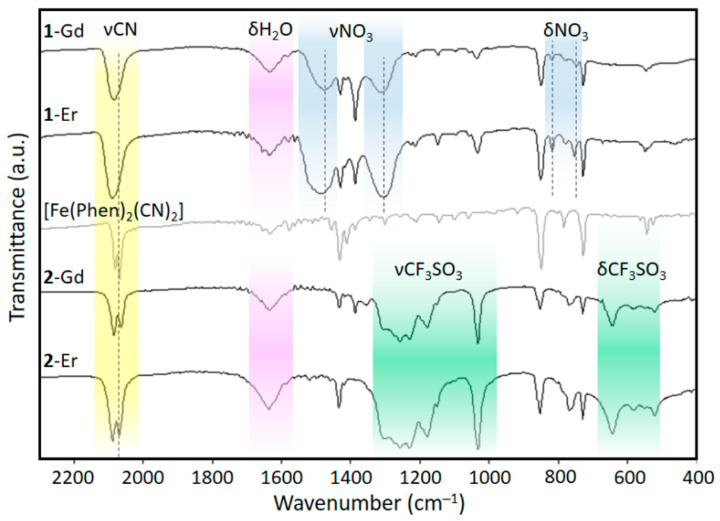
FT-IR spectra of compounds **1**-Ln and **2**-Ln (Ln = Gd, Er) compared with the precursor [Fe(Phen)_2_(CN)_2_].

The FT-IR spectra of **1**-Ln and **2**-Ln are reported in Figure 3 and compared to the vibrational spectrum of the precursor [Fe(Phen)_2_(CN)_2_]. The spectra of the isostructural gadolinium and erbium derivatives are also compared for both **1**-Ln and **2**-Ln to point out the sensitivity of vibrational modes to the nature of the metal ion. The bands of the phenanthroline ring are found for all compounds in the region 1430–1380 cm^−1^, corresponding to C=C and C=N in-plane modes, and in the region 900–700 cm^−1^, where the peaks of the out-of-plane vibrations of C-H groups fall. Interestingly, the relative intensity and the position of such bands related to the ancillary Phen ligand change to some extent upon coordination of the iron(II) precursor complex to the Ln ions and also based on the coordinated anion. This effect is likely ascribable to the change in the supramolecular arrangement of the complexes, where the π-stacking interactions significantly differ in **1**-Ln, **2**-Ln and [Fe(Phen)_2_(CN)_2_]·2H_2_O·CH_3_CN (Figure 2b–d). In the spectra of **1**-Gd and **1**-Er, the band related to the degenerate anti-symmetric stretching mode of NO_3_^−^ (ν_as_) splits into two main broad bands around ~1480 and 1310 cm^−1^ as a result of the bidentate coordination to the Ln ions. These bands show a certain sensitivity to the nature of the coordinated metal, displaying opposite shifts in wavenumber on increasing the atomic number (1473 and 1310 cm^−1^ for Gd; 1487 and 1307 cm^−1^ for Er). A medium-weak peak at 1030 cm^−1^ is attributed to the symmetric stretching (ν_s_) of the nitrate ion. The two small peaks around ~815 cm^−1^ and ∽745 cm^−1^ are attributed to the out-of-plane and the anti-symmetric bend (δ) of NO_3_^−^, respectively. This latter peak is particularly influenced by the nature of the coordinated Ln ion (744 cm^−1^ in **1**-Gd and 748 cm^−1^ in **1**-Er). In **2**-Gd and **2**-Er, the bands related to the triflate anion dominate the regions 1350–1000 cm^−1^ (symmetric and anti-symmetric stretching modes) and 650–500 cm^−1^ (bending modes). In this case, the CF_3_SO_3_^−^ anion coordinates in a monodentate fashion and no influence of the atomic number on the bonded Ln ion is detected in the vibrational spectra. The strong peak related to the symmetric and anti-symmetric stretching modes of the cyanide group appears around 2080 cm^−1^ in all compounds [30]. Interestingly, this peak is clearly split in **2**-Ln, similar to what was observed in the precursor [Fe(Phen)_2_(CN)_2_]·2H_2_O·2CH_3_CN, whereas no distinguishable fine structure is visible in the spectra of **1**-Ln. Ruling out the role of the solvent, since no CH_3_CN crystallization molecule is present in **2**-Ln, and considering that all cyanide groups are involved in bonding, we infer that this feature could be attributable to the difference in the coordination angles of the CN-Ln bridges in **1**-Ln (164°) and **2**-Ln (154°), with a more distorted arrangement with respect to a linear geometry found in the latter, as evidenced in Figure 2a. For comparison, the analogous compounds {Dy(CH_3_OH)_4_[Fe(CN)_2_(Phen)_2_]_2_} and {Dy(pyNO)_2_(CH_3_OH)_2_[Fe(CN)_2_(Phen)_2_]_2_} reported in reference [4] display CN-Dy angles of 176° and 168°, respectively, and no fine structure in the CN stretching IR band. In [Fe(Phen)_2_(CN)_2_]·2H_2_O·2CH_3_CN, the cyanide groups interact through hydrogen bonding with two water molecules and the CN···H angle is 165° (Figure S6) similar to **1**-Ln; most likely indicating that, in this case, the interaction with hydrogen is not strong enough to influence the CN stretching. A significant blue shift is observed upon coordination with the Ln ions, which is slightly more pronounced for erbium than gadolinium, in agreement with a possible electron density donation from an anti-bonding orbital of the cyanide N donor to the Ln ion (vide infra) [31].

### 2.3. Thermal Stability

**Figure 4:**
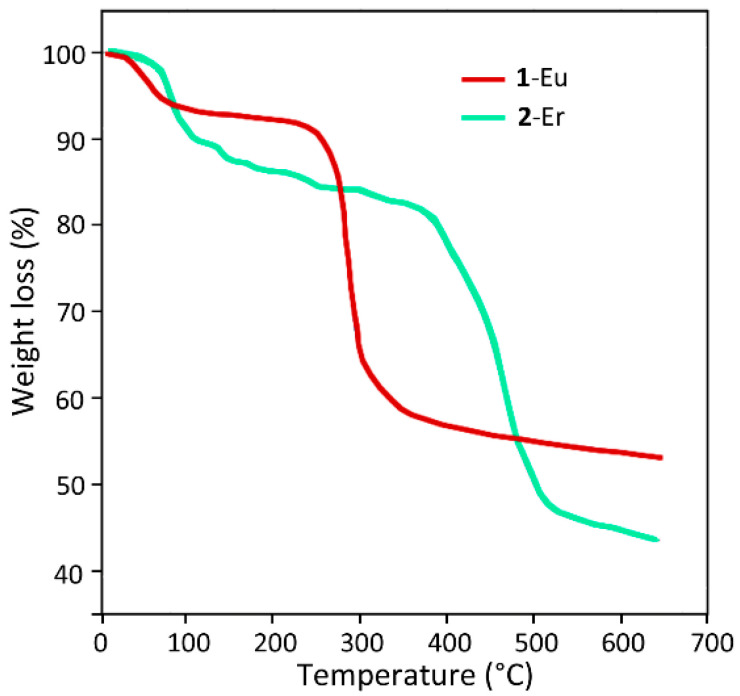
Thermogravimetric curves for **1**-Eu (red) and **2**-Er (green).

To assess the influence of the structural assembly on the thermal stability of the compounds, we performed thermal gravimetric analysis on **1**-Eu and **2**-Er as representative samples (Figure 4).

Both samples show a similar overall trend, with a first limited weight loss at lower temperatures and a more important loss at higher temperatures, in accordance with their analogous stoichiometry. In particular, the first limited weight loss (around 6.5% and 8.8% for **1**-Eu and **2**-Er, respectively) is observed up to 100 °C and is likely due to the loss of water molecules and solvent of crystallization in the two compounds. However, the most conspicuous difference is the main weight loss at higher temperatures, which can be deemed as the start of sample decomposition. In the case of the oligomer **1**-Er, this dominant weight loss leads to a weight loss of around 40% and is relatively sharp, being centered around 290 °C.

Instead, for the polymeric compound at temperatures above 100 °C, an overall weight loss of around 47% is recorded, which takes place through a more complex trend, including a smoother limited weight loss up to around 330 °C followed by a significant weight loss in the 400–500 °C range, mainly at ≈470 °C. These results point out the remarkable impact of the structural arrangement on the thermal stability of the compounds, where thermal analysis suggests a significantly higher stability for the polymeric assembly **2**-Er as compared to the oligomeric counterpart **1**-Eu. For comparison, the analogous compounds reported in reference [5] display thermal decomposition around 200 °C.

### 2.4. Optical Properties and Chromism

**Figure 5:**
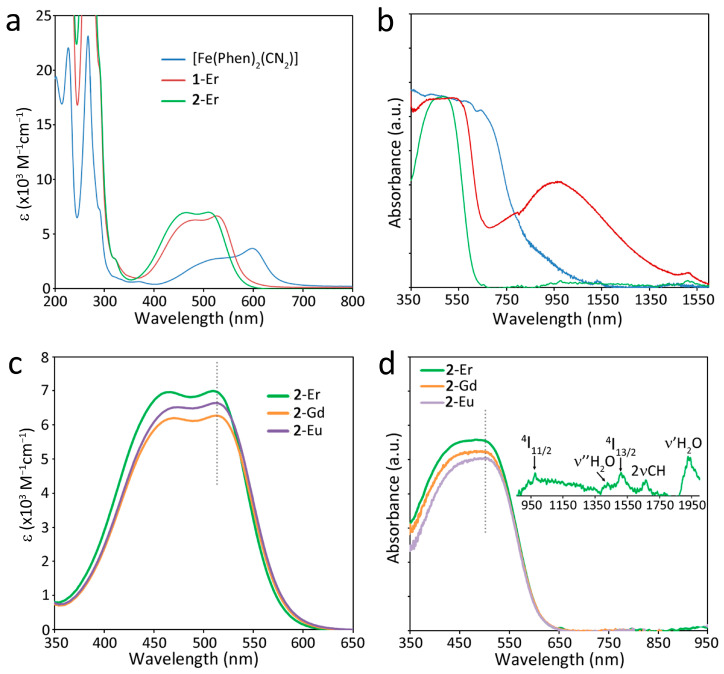
(**a**) Electronic absorption in acetonitrile solution and (**b**) diffuse reflectance spectra of crystalline samples of **1**-Er, **2**-Er and [Fe(Phen)_2_(CN)_2_] in the UV-Vis-NIR range. (**c**,**d**) Enlargement of the lowest energy absorption bands of **2**-Eu, **2**-Gd and **2**-Er in acetonitrile solution and in the solid state, respectively. The inset in (d) reports the NIR spectrum of **2**-Er showing the significant spectral features related to Er(III) absorption and the vibrational overtones of water and CH groups.

The precursor metalloligand [Fe(Phen)_2_(CN)_2_] is known to display remarkable solvatochromic and ionochromic behaviors and has been investigated in the past for its sensing properties toward solvent polarity and acidity [32,33,34] and cations [22,35]. The notable chromism of this complex is immediately evidenced by the visible change in color from dark purple to red upon coordination with the Ln ions in **1**-Ln and **2**-Ln both in acetonitrile solution and as crystalline samples. This is confirmed by solution and solid-state diffuse reflectance electronic spectra reported in Figure 5a,b, respectively, which show an evident blue shift of the lowest energy absorption in the visible range for the heterometallic compounds, also indicating that the heterometallic assembly is maintained in solution. In acetonitrile solution, the spectral shape is similar for all compounds, indicating that the absorption features are mainly related to the dicyano-bis(1,10-phenanthroline) iron(II) moiety and undergo a change in energy because of interactions through the cyanide groups. Two convolved bands are distinguishable in the visible region, displaying a blue shift that is significantly more accentuated in **2**-Ln (515 and 461 nm for **2**-Er) than **1**-Ln (525 and 471 nm for **1**-Er) with respect to [Fe(Phen)_2_(CN)_2_] (600 and 520 nm), Figure 5a. An identical trend is reproduced for crystalline samples (Figure 5b), even though the visible absorption band is broadened and red-shifted to some extent (the shift is less evident for **2**-Ln, Figure S7), a typical effect for solid-state samples. Since the coordinated Ln ion is the same, it can be reliably suggested that the observed spectral difference between **1**-Ln and **2**-Ln is essentially related to the different structural arrangements of the two compounds. In fact, when comparing the influence of the nature of the Ln ion on the spectral properties, only small deviations are found in the position of the lowest energy absorption band, where the blue shift follows the order Er > Gd > Eu, in accordance with the variation of the atomic number, as evidenced in Figure 2c,d for solution and solid-state samples, respectively. These observations further support the conclusion that the heterometallic structures are preserved in solution, but, more importantly, they point out the dominant role of the geometric architecture over the nature of the chemical species bonded to the cyanide groups.

The influence of the structural arrangement on the optical features of these compounds is also evidenced by the presence of an intense and broad (600–1500 nm) band in the NIR region in the spectrum of **1**-Ln (Figure 5b and Figure S8), which can be ascribed to a charge-transfer (CT) transition, likely involving phenanthroline rings that are π-stacked in the crystal packing. As shown in Figure 2, the π-stacking interactions seem more extensive and asymmetric in **1**-Ln with respect to **2**-Ln and [Fe(Phen)_2_(CN)_2_]·2H_2_O·2CH_3_CN, justifying the absence of such CT broad band in the spectra of the latter.

**Figure 6:**
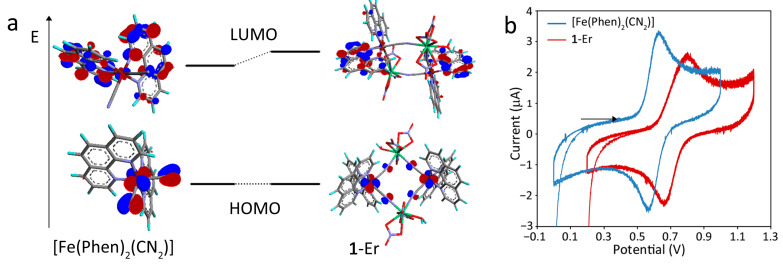
(**a**) DFT-calculated frontier molecular orbitals for [Fe(Phen)_2_(CN)_2_] (**left**) and **1**-Ln (**right**). (**b**) Cyclic voltammograms of [Fe(Phen)_2_(CN)_2_] and **1**-Ln in CH_3_CN solution against Ag/AgCl electrode. The arrow indicates the scan direction.

The optical chromism of the complex [Fe(Phen)_2_(CN)_2_] related to the lowest energy visible absorption band was traditionally associated with a metal-to-ligand charge-transfer (MLCT) transition involving the *d* orbitals of the Fe(II) ion and the π* orbitals of the phenanthroline ring [4,22,32]. Density Functional Theory (DFT) calculations (Tables S4–S6) have instead shown that such optical feature is rather associated with a transition from a highest occupied molecular orbital (HOMO), which is contributed by both the Fe(II) *d* orbitals and the π orbitals of the cyanide group, to a lowest occupied molecular orbital (LUMO) which is mainly localized on π orbitals of the overall anti-bonding character of the phenanthroline ring, as shown in Figure 2a, in agreement with previously reported theoretical studies [36]. Upon interaction with the Ln ion, the HOMO still retains the mixed *d* metal/cyanide ligand character, but the orbital density of the CN^−^ group becomes more localized on the *p* orbital of the nitrogen donor atom (Figure 6a). This may explain the observed strong negative ionochromism, which is likely related to the stabilization of the mixed Fe(II)(*d*)/CN N(*p*) HOMO due to the interaction with the positively charged Ln ion.

These considerations are in agreement with the observed shift toward a more positive potential for the Fe(II) ⟶ Fe(III) oxidation process in the heterometallic compound (E_1/2_ = +0.604 V to +0.731 V), as a result of the stabilization of the HOMO, which has a relevant contribution of the iron *d* orbitals (Figure 6b).

## 3. Materials and Methods

### 3.1. Materials and Analytical Measurements

All reactants and solvents were purchased from Sigma-Aldrich (Sigma-Aldrich, Saint-Louis, MO, USA) or Merck (Merck KGaA, Whitehouse Station, NJ, USA) and used without further purification. *Elemental analysis*: Data were collected with a Carlo Erba (Carlo Erba SpA, Milano, Italia) EA1108 CHNS analyzer. *Electronic Spectroscopy UV-Vis-NIR*. Diffuse reflectance (DR) and absorption spectra in CH_3_CN solution (10^−4^ mol/L) were collected with a Agilent Cary 500 spectrophotometer (Agilent Technologies, Santa Clara, CA, USA) equipped with a 150 mm diameter integrating sphere. Crystalline samples for DR spectra were dispersed on a Teflon film. *Vibrational Spectroscopy*. FT-IR spectra on KBr pellets were collected with a Bruker Equinox 55 spectrophotometer. *Cyclic Voltammetry*. Cyclic voltammetry experiments were performed at room temperature on 10^−4^ M CH_3_CN solutions with an AUTOLAB PGSTAT302N (Metrohm, Herisau, Switzerland) potentiostat/galvanostat controlled with the NOVA software, at a scan rate of 100 mV/s. Ag/AgCl(sat.) was used as the counter-electrode and tetrabutylammonium hexafluorophosphate 0.1 M as the electrolyte. Solutions were fluxed with Ar prior to each measurement. *Thermal analysis*: Thermal analysis was performed on an SDT Q600 instrument (TA Instruments, New Castle, DE, USA) operating under N_2_ (flow rate 50 mL∙min^−1^). The runs were collected at a heating rate of 10 °C∙min^−1^ up to 650 °C. *Powder-XRD:* Powder-XRD patterns were collected in the 2θ range from 5° to 50° (Cu Kα) by using a Bruker D8 Advance diffractometer (Bruker AXS, Billerica, MA, USA).

### 3.2. Syntheses

*[Fe(phen)_2_(CN)_2_]·2H_2_O·CH_3_CN:* Fe(NH_4_)_2_(SO_4_)_2_·6H_2_O (0.975 g, 2.5 mmol) and 1,10-phenatroline (1.5 g, 7.5 mmol) were dissolved in 100 mL of distilled water, and the dark red mixture was heated to just below the boiling point. Afterward, a freshly prepared solution of KCN (2.5 g) in 5 mL of H_2_O was added all at once. After 30′ of stirring, the reaction mixture was allowed to cool down to room temperature, and purple crystalline platelets began to form. After 24 h, the crystalline product was filtered, washed with water and dried in a vacuum at room temperature. Dark-violet crystals suitable for X-ray studies were obtained from recrystallization in CH_3_CN. Elemental analysis: Data found (calculated for C_28_H_23_FeN_7_O_2_): C% 62.08(61.66), H% 4.64(4.25) and N% 17.56(17.98).

***1****-Ln:* [Fe(phen)_2_(CN)_2_]·2H_2_O·CH_3_CN (0.123 g, 0.243 mmol) was dissolved in 100 mL of CHCl_3_/CH_3_CN 1:1 solvent mixture under gentle heating. When all the solid was dissolved, the resulting dark violet solution was cooled down in an ice bath, and 20 mL of an equimolar solution of Ln(NO_3_)_3_·nH_2_O (Ln = Eu, n = 6; Gd n = 6; Er, n = 5) in CH_3_CN was slowly added. The reaction mixture turned suddenly red. After a few minutes, red crystals began to form and the mixture was left standing overnight at 4 °C. Red crystals suitable for X-ray studies were collected, washed with CHCl_3_ to dissolve all the unreacted iron complex precursor and dried in a vacuum at room temperature. The yield was 75%. Elemental analysis: Data found (calculated for C_28_H_21_FeLnN_10_O_10_): **1**-Eu C% 38.39 (38.86), H% 2.58 (2.45) and N% 16.24 (16.19); **1**-Gd C% 38.80 (38.63), H% 2.66 (2.43) and N% 16.11 (16.09); **1**-Er C% 38.02 (38.19), H% 2.63 (2.40) and N% 16.04 (15.91).

***2****-Ln:* These compounds were synthesized with the same procedure described for 1-Ln except that triflate salts Ln(CF_3_SO_3_)_3_·nH_2_O (Ln = Eu, n = 6; Gd n = 6; Er, n = 5) were used in place of the nitrate salts. The yield was 82%. Elemental analysis: Data found (calculated for C_29_H_28_F_9_FeLnN_6_O_15_S_3_): **2**-Eu C% 30.19 (29.63), H% 2.58 (2.40), N% 7.24 (7.15) and S% 8.17 (8.18); **2**-Gd C% 30.01 (29.50), H% 2.36 (2.39), N% 7.12 (7.12) and S% 8.10 (8.15); **2**-Er C% 29.75 (29.25), H% 2.79 (2.37), N% 7.11 (7.06) and S% 8.07 (8.08). It should be mentioned that severely disordered CHCl_3_ crystallization molecules were found from single-crystal XRD data. However, in light of the fact that these highly volatile solvent molecules can be easily lost by filtration or sample manipulation, and considering the better agreement of data, we calculated elemental composition and reaction yields without taking into account possible CHCl_3_ co-crystallization molecules.

### 3.3. X-ray Crystallography

Single crystal data were collected with a *Bruker AXS Smart 1000* (Bruker AXS, Billerica, MA, USA) (**1**-Eu), a *Philips PW 1100* (Philips, Amsterdam, Netherlands) (**1**-Er) and *Bruker Smart APEXII* diffractometers Bruker AXS, Billerica, MA, USA) (**2**-Gd, **2**-Eu, **2**-Er). All data collection were performed with the Mo Kα radiation (λ = 0.71073 Å). Cell constants of **1**-Er were obtained by a least-square refinement of the setting angles of 24 randomly distributed and carefully centered reflections (5.65 < 2*θ* < 14.70), whereas the unit cell parameters of **1**-Eu and **2**-Gd, **2**-Eu, **2**-Er were obtained using 60 ω-frames of 0.5° width and scanned from three different zones of the reciprocal lattice. The intensity data of **1**-Eu and **2**-Gd, **2**-Eu, **2**-Er were integrated from several series of exposure frames (0.3° width) covering the sphere of reciprocal space [37]. An absorption correction was applied for **1**-Eu and **2**-Gd, **2**-Eu, **2**-Er using the program SADABS [38] with min. and max. transmission factors of 0.701–1.000 (**1**-Eu), 0.741–1.000 (**2**-Gd), 0.826–1.000 (**2**-Eu) and 0.817–1.000 (2-Er), respectively. For **1**-Er, an empirical absorption correction was applied using the program NEWABS92 [39] (min. and max. transmission factors of 0.868–1.000). The structures were solved by direct methods (SIR97 [40]) and refined on *F*^2^ with full-matrix least squares (SHELXL-97 [41]), using the Wingx software package [42]. Compound **1**-Eu is isostructural with **1**-Er, whereas **2**-Gd is isostructural with **2**-Eu and **2**-Er. In **2**-Gd, one CHCl_3_ molecule could be located from the difference Fourier map and was found disordered in two positions, each with a site occupancy factor of 0.5. Another CHCl_3_ solvent molecule was severely disordered and was modeled with the SQUEEZE program [43], which gave a void volume of 936 Å^3^ and 180 electrons per unit cell. In **2**-Er and **2**-Eu, both CHCl_3_ solvent molecules were modeled with the SQUEEZE program, which gave a void volume of 1536 Å^3^ and 380 electrons per unit cell for **2**-Er, and a void volume of 1544 Å^3^ and 436 electrons per unit cell for **2**-Eu.

Non-hydrogen atoms were refined anisotropically for all compounds, except for the CHCl_3_ molecules in **2**-Gd, **2**-Eu and **2**-Er. The hydrogen atoms of the water molecules of all compounds were located from the difference Fourier map, whereas the remaining hydrogen atoms were placed at their calculated positions. Graphical material was prepared with the ORTEP3 for Windows [44] and Mercury CSD 2.0 [45] programs. CCDC 2245337–2245342 contain the supplementary crystallographic data for this paper.

### 3.4. DFT Calculations

Electronic structure calculations were performed at the DFT [46] level employing the GAUSSIAN 16 [47] software package. The functional used throughout this study was CAM-B3LYP [48], with basis set 6–31 + G(d,p) [49] for C, H, O, N, S and F atoms, and the LANL2DZ [50] and SDD [51] ECP basis sets for Fe and Er, respectively. All calculations were input using atomic coordinates obtained from single X-ray data. The orbital isosurfaces (with isovalue plot 0.04) were visualized using ArgusLab 4.0 [52].

## 4. Conclusions

A rare example of anion-induced structural diversity in trivalent lanthanide-based heterometallic complexes has been explored. Cyano-bridged 3*d*-4*f* compounds have been obtained by a direct self-assembly reaction of lanthanide nitrate and triflate salt with the neutral metalloligand [Fe(Phen)_2_(CN)_2_]. While isostructurality is observed by the reaction of either “light” or “heavy” lanthanide ions, it has been found that the triflate anion used in place of the nitrate has dramatic consequences on the structure and shape of the resulting complexes, under identical synthetic conditions. In fact, the bidentate nitrate anion leads to the formation of tetranuclear structures with a Fe_2_Ln_2_ core of squared topology with only one water molecule in the lanthanide coordination sphere. On the other hand, the more sterically demanding triflate acts as O-monodentate toward the lanthanide ion, thus leaving several coordination positions open for the coordination of water molecules, which are in turn involved in an extensive net of hydrogen bonds, resulting in an unusual 1D coordination polymer. The different structural motifs have important consequences on the thermal stability of the compounds, where the polymeric assembly starts to decompose at much higher temperatures than the oligomer. A remarkable negative optical chromism is observed for both classes of heterometallic compounds with respect to the iron(II) metalloligand, accompanied by an evident color change from dark purple to red both in acetonitrile solution and in the solid state. Unexpectedly, the extent of the spectral shift displayed by the lowest energy band in the visible range, corresponding to a HOMO-LUMO transition, is significantly more accentuated in the coordination polymer than in the tetranuclear oligomer when coordinated to the same lanthanide ion. Therefore, the structural arrangement of the heterometallic architecture, which is preserved upon dissolution in acetonitrile, has a much more significant influence on the observed optical properties over the nature of the coordinated lanthanide ion, which, in turn, has a small effect. Structural evidence suggests that this behavior is likely related to the geometry of the CN-Ln bridge, which significantly deviates from linearity in the polymeric assembly. DFT calculations, supported by electrochemical studies, demonstrate that the negative ionochromism is related to a stabilization of the HOMO, which has a mixed character consisting of the contributions of Fe(II) *d* orbitals and anti-bonding cyano N *p* orbitals, whereas the LUMO is mostly a π* orbital located on the phenanthroline ring. Interestingly, the tetranuclear compound displays a broad NIR charge-transfer band in the solid state, which is likely related to extensive π-stacking interactions among phenanthroline ligands, which is not present in the spectra of the polymer as well as the Fe(II) precursor.

These results provide novel perspectives on the architectural construction of heterometallic 3*d*-4*f* compounds that are of current interest for the development of mono- and multi-functional magnetic/optical materials, including single-molecule magnets and chemical sensors. In such systems, the replacement of coordinated water molecules by suitable chelating agents/organic ligands also represents a great opportunity to expand and improve their functionalities and application potential.

Novel insights on the long-known optical chromism properties of the [Fe(Phen)_2_(CN)_2_] complex have also been highlighted in this work.

## Figures and Tables

**Table 1:** Summary of X-ray crystallographic data for **1**-Ln.

	1-Eu	1-Er
Empirical formula	C_56_H_42_Eu_2_Fe_2_N_20_O_20_	C_56_H_42_Er_2_Fe_2_N_20_O_20_
Formula weight	1730.72	1761.32
Color, habit	Prism, red	Prism, red
Crystal size, mm	0.40 × 0.30 × 0.18	0.20 × 0.10 × 0.05
Crystal system	Monoclinic	Monoclinic
Space group	*P*2_1_/a	*P*2_1_/a
*a*, Å	10.730(1)	10.74(7)
*b*, Å	25.623(3)	25.59(9)
*c*, Å	11.789(2)	11.74(8)
α, deg.	90	90
β, deg.	100.63(2)	100.26(3)
γ, deg.	90	90
*V*, Å^3^	3181.6(7)	3174(3)
*Z*	2	2
*T*, K	293(2)	293(2)
*ρ* (calc), Mg/m^3^	1.804	1.843
*μ*, mm^−1^	2.479	3.256
*θ* range, deg.	1.59 to 28.02	3.07 to 25.00
No. of rflcn/obsv	39,388/5692	6741/2582
GooF	1.002	0.768
*R*1	0.0299	0.0482
*wR*2	0.0528	0.0535

R1 = Σ||Fo|-|Fc||/Σ|Fo|, wR2 = [Σ[w(Fo2-Fc2)2]/Σ[w(Fo2)2]]½, w = 1/[σ2(Fo2) + (aP)2 + bP], where P = [max(Fo2,0) + 2Fc2]/3.

**Table 2:** Summary of X-ray crystallographic data for **2**-Ln.

	2-Gd	2-Eu	2-Er
Empirical formula	C_30_H_29_Cl_3_F_9_FeGdN_6_O_15_S_3_	C_30_H_29_Cl_3_EuF_9_FeN_6_O_15_S_3_	C_30_H_29_Cl_3_ErF_9_FeN_6_O_15_S_3_
Formula weight	1300.22	1294.93	1310.23
Color, habit	Plate, orange	Plate, orange	Plate, orange
Crystal size, mm	0.45 × 0.22 × 0.10	0.15 × 0.08 × 0.05	0.12 × 0.07 × 0.05
Crystal system	Monoclinic	Monoclinic	Monoclinic
Space group	*C*2/c	*C*2/c	*C*2/c
*a*, Å	33.191(2)	33.158(4)	33.247(8)
*b*, Å	13.790(1)	13.809(2)	13.716(3)
*c*, Å	27.296(1)	27.281(3)	27.173(5)
α, deg.	90	90	90
β, deg.	131.359(1)	131.197(2)	131.16(3)
γ, deg.	90	90	90
*V*, Å^3^	9378.8(9)	9399(2)	9329(3)
*Z*	8	8	8
*T*, K	293(2)	293(2)	293(2)
*ρ* (calc), Mg/m^3^	1.842	1.830	1.866
*μ*, mm^−1^	2.120	2.039	2.508
*θ* range, deg.	1.53 to 31.10	1.53 to 25.09	1.53 to 24.90
No. of rflcn/obsv	76,817/11,455	50,971/5560	48,135/5383
GooF	1.020	1.051	1.015
*R*1	0.0433	0.0556	0.0677
*wR*2	0.1322	0.1299	0.1663

R1 = Σ||Fo|-|Fc||/Σ|Fo|, wR2 = [Σ[w(Fo2-Fc2)2]/Σ[w(Fo2)2]]½, w = 1/[σ2(Fo2) + (aP)2 + bP], where P = [max(Fo2,0) + 2Fc2]/3.

## Data Availability

The data presented in this study are available on request from the corresponding authors.

## Supplementary Materials

The following supporting information can be downloaded at https://www.mdpi.com/article/10.3390/molecules28062871/s1, Figure S1: ORTEP drawing of [Fe(Phen)_2_(CN)_2_]·2H_2_O·CH_3_CN; Figure S2: Capped square anti-prismatic geometry of the lanthanide ion in 1-Eu; Figure S3: Square anti-prismatic geometry of the lanthanide ion in 2-Gd; Figure S4: Portion of the crystal packing of 2-Eu; Figure S5: Powder-XRD patterns; Figure S6: Schematic representation of the CN···H angle in [Fe(Phen)_2_(CN)_2_]·2H_2_O·CH_3_CN; Figure S7: Electronic absorption of CH_3_CN solutions and solid-state diffuse reflectance spectra of 1-Er and 2-Er compared with the precursor [Fe(Phen)_2_(CN)_2_] complex; Figure S8: Solid-state diffuse reflectance spectra of crystalline samples of 1-Ln (Ln = Er, Gd, Eu); Table S1: Summary of X-ray crystallographic data for [Fe(Phen)_2_(CN)_2_]·2H_2_O·CH_3_CN; Table S2: Selected bond lengths (Å) and angles (°) for 1-Eu and 1-Er; Table S3: Selected bond lengths (Å) and angles (°) for 2-Ln; Table S4: DFT-calculated molecular orbitals of [Fe(phen)_2_CN_2_]; Table S5: DFT-calculated molecular orbitals of 1-Er; Table S6: DFT-calculated molecular orbitals of 2-Er.

## Sample Availability

Samples of the reported compounds are available from the authors upon reasonable request.
