# Supplementary material for: Anion-Induced Structural Diversity and Optical Chromism in a Series of Cyano-Bridged Heterometallic 3d-4f Coordination Polymers
Source: Molecules. 2023 Mar 22;28(6):2871. doi: 10.3390/molecules28062871 (PMC10056676; doi:10.3390/molecules28062871)
Supplement: Supplementary file 1 [file molecules-28-02871-s001.zip › molecules-2289162-supplementary.pdf]

Anion-induced Structural Diversity and Optical Chromism in a Series of Cyano-bridged Heterometallic 3*d*-4*f* Coordination Polymers

Flavia Artizzu, Luca Pilia, Angela Serpe, Dimitrije Mara, Maria Francesca Casula, Luciano Marchiò and Paola Deplano

Supporting Information

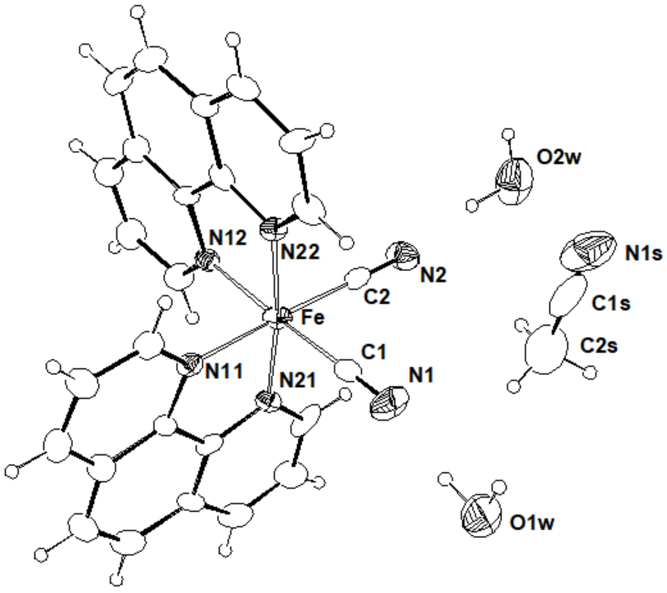

Figure S1. Ortep drawing of $[Fe(Phen)_2(CN)_2]{\cdot}2H_2O{\cdot}CH_3CN$. Ellipsoids are drawn at 30% probability level.

Table S1. Summary of X-ray crystallographic data for $[Fe(Phen)_2(CN)_2]{\cdot}2H_2O{\cdot}CH_3CN$.

Empirical formula	$C_{28}H_{23}FeN_7O_2$
Formula weight	545.38
Colour, habit	Prisms, purple
Crystal size, mm	0.10×0.08×0.05
Crystal system	Triclinic
Space group	*P*-1
a, Å	10.266(4)
b, Å	10.396(4)
c, Å	12.107(4)
α, deg.	80.92(1)
β, deg.	78.75(1)
γ, deg.	87.07(1)
V, Å^3	1251.1(8)
Z	2
T, K	293(2)
ρ (calc), Mg/m^3	1.448
μ, mm^{-1}	0.644
θ range, deg.	1.73 to 24.04
No.of rflcn/obsv	39388 / 5692
GooF	0.892
*R*1	0.0555
*wR*2	0.0584

$R1 = \Sigma||F_o|-|F_c||/\Sigma|F_o|$, $wR2 = [\Sigma[w(F_o^2-F_c^2)^2]/\Sigma[w(F_o^2)^2]]^{½}$, $w = 1/[\sigma^2(F_o^2) + (aP)^2 + bP]$, where $P = [\max(F_o^2,0) + 2F_c^2]/3$

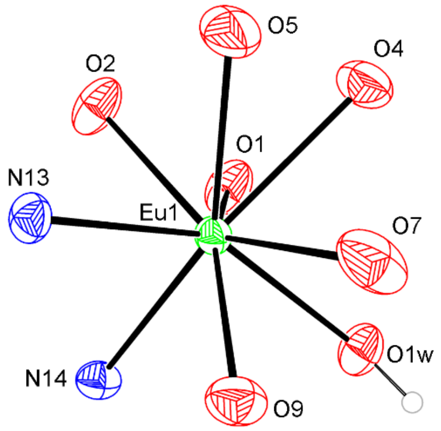

Figure S2. Capped square anti-prism geometry of the lanthanide ion in **1**-Eu. The square faces are represented by O(2)-O(4)-N(14)-O1w and by O(5)-O(7)-N(13)-O(9); O(1) is capping the former face.

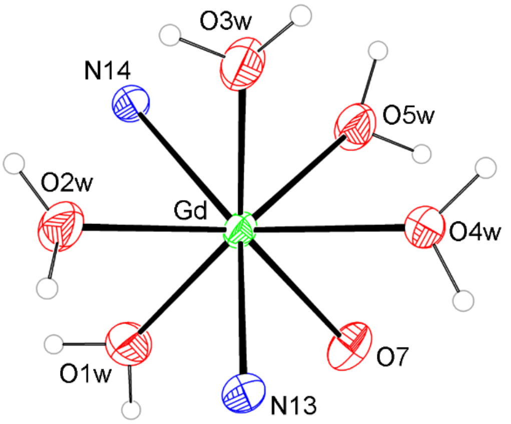

Figure S3. Square anti-prism geometry of the lanthanide ion in **2**-Gd. The square faces are represented by O1w-N(14)-O5w-O(7) and by O2w-O3w-O4w-N(13).

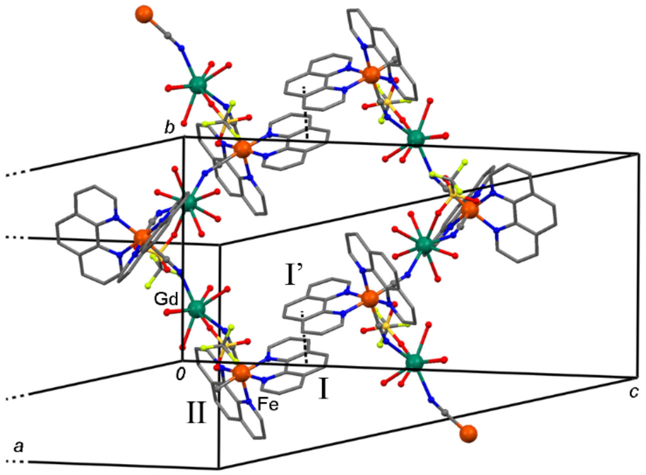

Figure S4. Portion of the crystal packing of **2**-Eu. Only the coordination environment of the metal is reported for clarity. The π-stacking between **I** phenanthroline rings are reported as dashed bonds. Symmetry code ′ = ½-x; ½-y; 1-z.

Table S2. Selected bond lengths (Å) and angles (°) for **1**-Eu and **1**-Er.

**1**-Eu			
Eu(1)-O(1)	2.538(2)	N(14)-Eu(1)-O(1)	75.03(8)
Eu(1)-O(2)	2.508(2)	N(14)-Eu(1)-O1w	78.68(9)
Eu(1)-O(4)	2.469(2)	O(1)-Eu(1)-O1w	70.09(8)
Eu(1)-O(5)	2.498(2)	N(13)-Eu(1)-O(5)	74.98(9)
Eu(1)-O(7)	2.480(3)	N(14)-Eu(1)-N(13)	83.05(8)
Eu(1)-O(9)	2.470(3)	O(1)-Eu(1)-O(5)	102.1(1)
Eu(1)-O1w	2.432(2)	O1w-Eu(1)-O(7)	75.8(1)
Eu(1)-N(13)	2.429(3)	N(13)-Eu(1)-O(2)	79.68(8)
Eu(1)-N(14)	2.482(2)	O(2)-Eu(1)-O(5)	74.7(1)
Fe(2)-N(11)	1.970(2)	O(5)-Eu(1)-O(4)	50.51(9)
Fe(2)-N(21)	1.996(2)	O(4)-Eu(1)-O(7)	70.92(9)
Fe(2)-N(12)	2.018(2)	O1w-Eu(1)-O(9)	79.80(9)
Fe(2)-N(22)	1.976(2)	O(9)-Eu(1)-N(14)	89.23(8)
Fe(2)-C(14)	1.911(3)	N(11)-Fe(2)-C(14)	94.4(1)
Fe(2)-C(13)′	1.901(3)	N(11)-Fe(2)-C(13)′	92.6(1)
Eu(1)-Fe(2)	5.5176(7)	N(22)-Fe(2)-C(14)	93.5(1)
Eu(1)-Fe(2)′	5.406(1)	N(22)-Fe(2)-C(13)′	91.4(1)
		N(12)-Fe(2)-C(13)′	91.9(1)
		N(21)-Fe(2)-C(14)	89.3(1)
		N(14)-C(14)-Fe(2)	176.7(3)
		N(13)′-C(13)′-Fe(2)	175.9(3)
		C(14)-N(14)-Eu(1)	169.4(2)
		C(13)-N(13)-Eu(1)	164.3(2)
**1**-Er			
Er(1)-O(1)	2.51(1)	N(14)-Er(1)-O(1)	74.7(4)
Er(1)-O(2)	2.45(1)	N(14)-Er(1)-O1w	79.1(4)
Er(1)-O(4)	2.41(1)	O(1)-Er(1)-O1w	69.8(4)
Er(1)-O(5)	2.44(1)	N(13)-Er(1)-O(5)	74.6(4)
Er(1)-O(7)	2.43(1)	N(14)-Er(1)-N(13)	82.2(4)
Er(1)-O(9)	2.41(1)	O(1)-Er(1)-O(5)	103.2(4)
Er(1)-O1w	2.36(1)	O1w-Er(1)-O(7)	76.3(4)
Er(1)-N(13)	2.37(1)	N(13)-Er(1)-O(2)	79.0(3)
Er(1)-N(14)	2.42(1)	O(2)-Er(1)-O(5)	73.9(5)
Fe(2)-N(11)	1.97(1)	O(5)-Er(1)-O(4)	51.7(3)
Fe(2)-N(21)	2.00(1)	O(4)-Er(1)-O(7)	71.0(3)
Fe(2)-N(12)	2.03(1)	O1w-Er(1)-O(9)	79.6(5)
Fe(2)-N(22)	1.98(1)	O(9)-Er(1)-N(14)	82.8(3)
Fe(2)-C(14)	1.93(1)	N(11)-Fe(2)-C(14)	94.9(4)
Fe(2)-C(13)′	1.91(1)	N(11)-Fe(2)-C(13)′	92.4(6)
Er(1)-Fe(2)	5.47(1)	N(22)-Fe(2)-C(14)	93.7(4)
Er(1)-Fe(2)′	5.36(1)	N(22)-Fe(2)-C(13)′	92.1(6)
		N(12)-Fe(2)-C(13)′	92.5(4)
		N(21)-Fe(2)-C(14)	88.8(4)

		N(14)-C(14)-Fe(2)	177(1)
		N(13)′-C(13)′-Fe(2)	175.3(9)
		C(14)-N(14)-Er(1)	169.0(9)
		C(13)-N(13)-Er(1)	166.0(8)

′ = -x; -y; 1-z

Table S3. Selected bond lengths (Å) and angles (°) for **2**-Ln.

**2**-Gd			
Gd-O1w	2.391(3)	O1w-Gd-O2w	76.4(1)
Gd-O2w	2.404(3)	O2w-Gd-N(14)	76.51(9)
Gd-O3w	2.398(3)	N(14)-Gd-O3w	78.6(1)
Gd-O4w	2.456(3)	O3w-Gd-O5w	84.1(1)
Gd-O5w	2.389(3)	O5w-Gd-O4w	76.7(1)
Gd-O(7)	2.417(3)	O4w-Gd-O(7)	76.7(1)
Gd-N(13)	2.426(3)	O(7)-Gd-N(13)	81.9(1)
Gd-N(14)	2.479(3)	N(13)-Gd-O1w	83.0(1)
Fe-N(11)	1.985(2)	N(13)-Gd-N(14)	147.07(9)
Fe-N(21)	2.012(3)	N(11)-Fe-C(14)′	94.0(1)
Fe-N(12)	2.006(3)	N(11)-Fe-C(13)	91.4(1)
Fe-N(22)	1.973(3)	N(22)-Fe-C(13)	93.4(1)
Fe-C(13)	1.884(3)	N(22)-Fe-C(14)′	92.5(1)
Fe-C(14)′	1.903(3)	N(12)-Fe-C(14)′	91.0(1)
Gd-Fe	5.2840(5)	N(21)-Fe-C(13)	92.9(1)
Gd-Fe′	5.4086(6)	N(13)-C(13)-Fe	174.6(3)
		N(14)′-C(14)′-Fe	178.4(3)
		C(13)-N(13)-Gd	153.1(2)
		C(14)-N(14)-Gd	156.1(2)
**2**-Eu			
Eu-O1w	2.411(6)	O1w-Eu-O2w	76.7(2)
Eu-O2w	2.412(5)	O2w-Eu-N(14)	77.8(2)
Eu-O3w	2.417(6)	N(14)-Eu-O3w	78.4(2)
Eu-O4w	2.475(5)	O3w-Eu-O5w	83.7(2)
Eu-O5w	2.384(5)	O5w-Eu-O4w	74.6(2)
Eu-O(7)	2.424(5)	O4w-Eu-O(7)	76.9(2)
Eu-N(13)	2.435(6)	O(7)-Eu-N(13)	81.6(2)
Eu-N(14)	2.494(6)	N(13)-Eu-O1w	82.7(2)
Fe-N(11)	1.974(5)	N(13)-Eu-N(14)	146.8(2)
Fe-N(21)	2.013(6)	N(11)-Fe-C(14)′	94.0(2)
Fe-N(12)	2.016(6)	N(11)-Fe-C(13)	91.5(2)
Fe-N(22)	1.965(6)	N(22)-Fe-C(13)	93.7(3)
Fe-C(13)	1.893(7)	N(22)-Fe-C(14)′	91.9(3)
Fe-C(14)′	1.888(7)	N(12)-Fe-C(14)′	91.0(2)
Eu-Fe	5.288(1)	N(21)-Fe-C(13)	93.0(2)
Eu-Fe′	5.414(1)	N(13)-C(13)-Fe	174.4(6)
		N(14)′-C(14)′-Fe	177.4(6)
		C(13)-N(13)-Eu	153.2(5)

		C(14)-N(14)-Eu	155.9(5)
**2**-Er			
Er-O1w	2.347(7)	O1w-Er-O2w	76.5(3)
Er-O2w	2.363(7)	O2w-Er-N(14)	75.9(3)
Er-O3w	2.405(6)	N(14)-Er-O3w	81.3(2)
Er-O4w	2.425(7)	O3w-Er-O5w	84.8(3)
Er-O5w	2.347(7)	O5w-Er-O4w	74.1(3)
Er-O(7)	2.373(7)	O4w-Er-O(7)	76.5(3)
Er-N(13)	2.361(8)	O(7)-Er-N(13)	81.7(3)
Er-N(14)	2.416(8)	N(13)-Er-O1w	83.7(3)
Fe-N(11)	1.970(8)	N(13)-Er-N(14)	147.1(3)
Fe-N(21)	2.025(8)	N(11)-Fe-C(14)′	95.1(3)
Fe-N(12)	2.002(8)	N(11)-Fe-C(13)	93.6(4)
Fe-N(22)	1.961(8)	N(22)-Fe-C(13)	93.8(4)
Fe-C(13)	1.89(1)	N(22)-Fe-C(14)′	91.1(3)
Fe-C(14)′	1.93(1)	N(12)-Fe-C(14)′	90.2(3)
Er-Fe	5.236(2)	N(21)-Fe-C(13)	93.6(3)
Er-Fe′	5.364(2)	N(13)-C(13)-Fe	174.1(8)
		N(14)′-C(14)′-Fe	174.7(8)
		C(13)-N(13)-Er	155.4(7)
		C(14)-N(14)-Er	156.8(7)

′ = ½-x; ½+y; ½-z

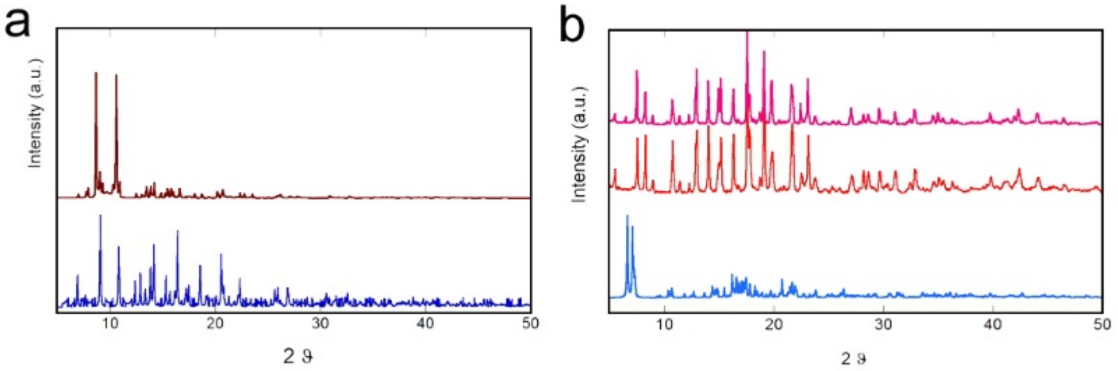

Figure S5. Powder-XRD patterns in the2θ range from 5° to 50° (Cu Kα). a) Experimental (above) and simulated (below) patterns of **1**-Er; b) Experimental patterns for **2**-Gd (pink), **2**-Eu (red) and simulated pattern of **2**-Eu (blue). Simulated patterns have been retrieved from single-crystal data. Some deviations of experimental data from the simulated ones in **2**-Ln are attributable to the loss of crystallization solvent over time and upon sample grinding.

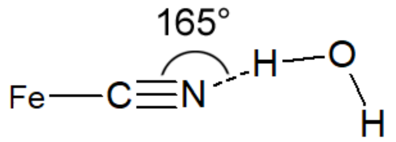

Figure S6. Schematic representation of the CN···H angle in $[Fe(Phen)_2(CN)_2]·2H_2O·CH_3CN$.

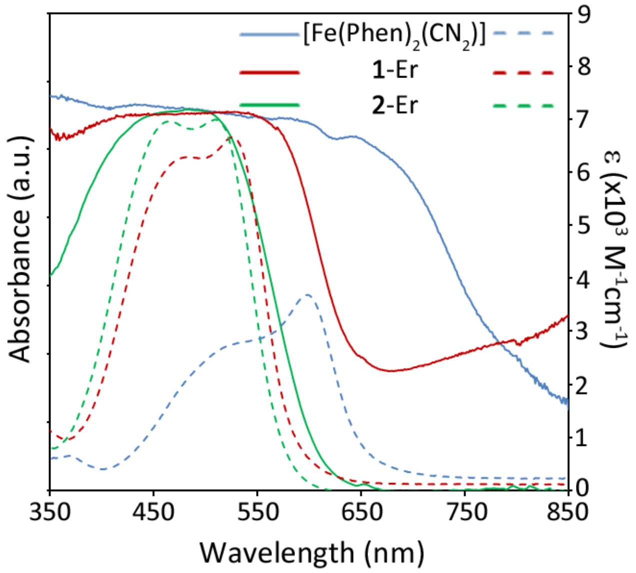

Figure S7. Electronic absorption of CH_3CN solutions (dashed lines) and solid-state diffuse reflectance (solid lines) spectra of **1**-Er and **2**-Er compared with the precursor $[Fe(Phen)_2(CN)_2]$ complex.

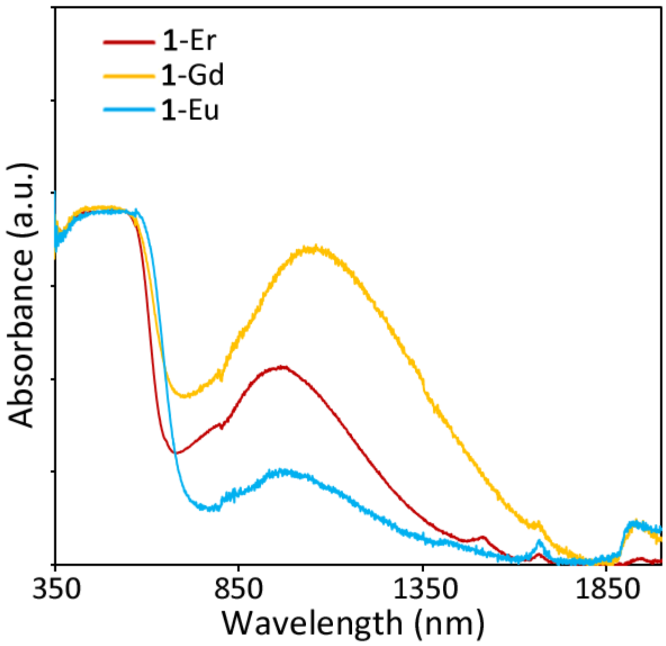

Figure S8. Solid-state diffuse reflectance spectra of crystalline samples of **1**-Ln (Ln = Er, Gd, Eu). The spectra are normalized for the absorption maximum in the visible range.

Table S4. DFT calculated molecular orbitals of [Fe(phen)$_2$CN$_2$] (isovalue plot 0.04).

LUMO+5<br>0.17	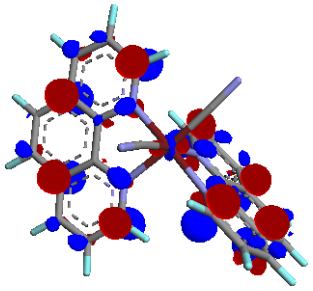	LUMO+4<br>-0.026	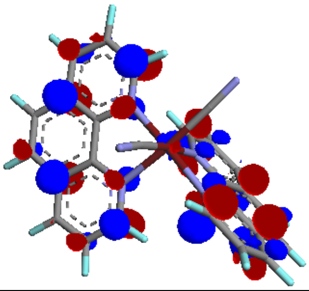
LUMO+3<br>-1.09	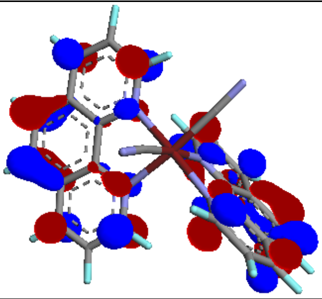	LUMO+2<br>-1.15	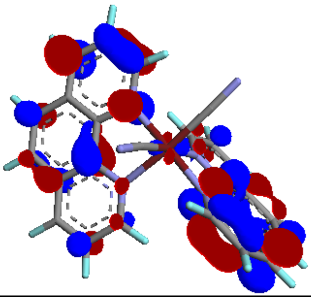
LUMO+1<br>-1.34	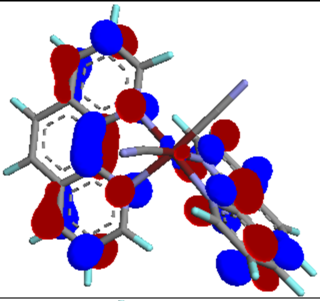	**LUMO**<br>-1.38	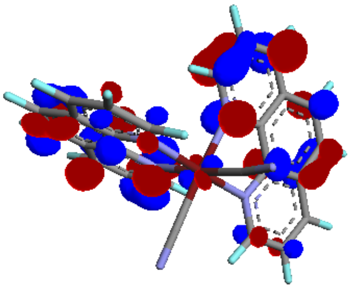
**HOMO**<br>**-6.74**	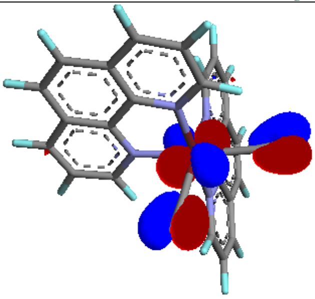	HOMO-1<br>-7.04	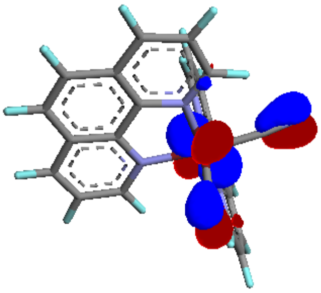
HOMO-2<br>-7.06	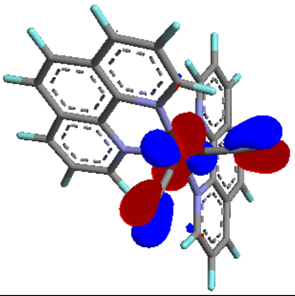	HOMO-3<br>-7.97	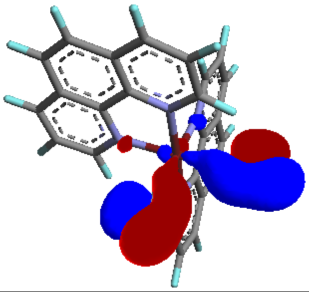
HOMO-4<br>-8.36	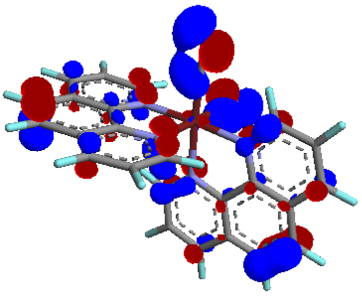	HOMO-5<br>-8.48	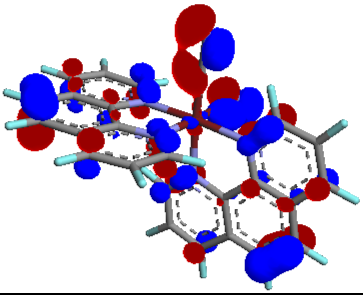

Table S5. DFT calculated molecular orbitals of **1**-Er (isovalue plot 0.04).

LUMO+5 -1.28	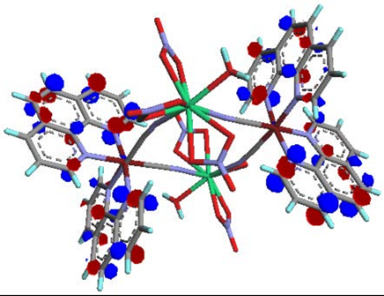	LUMO+4 -1.28	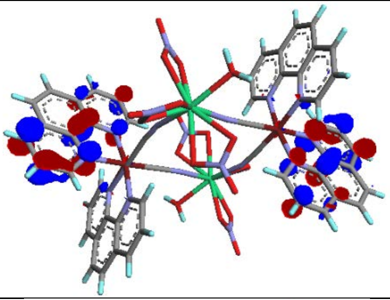
LUMO+3 -1.52	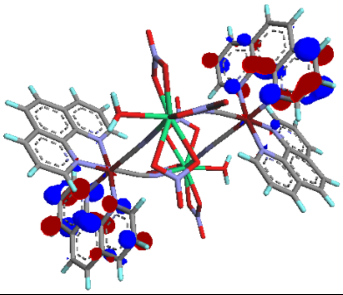	LUMO+2 -1.52	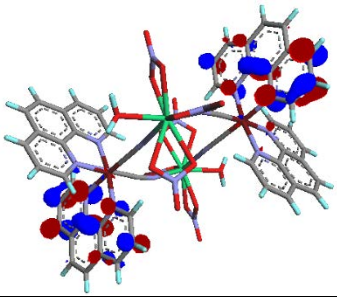
LUMO+1 -1.58	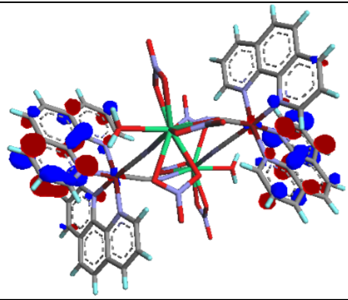	**LUMO -1.58**	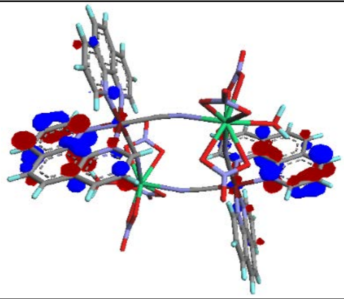
**HOMO -7.58**	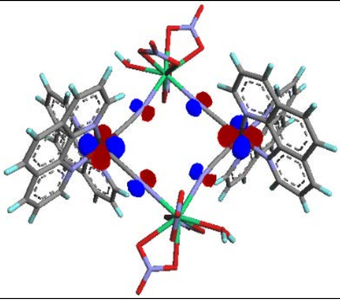	HOMO-1 -7.65	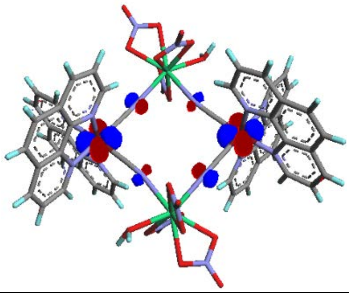
HOMO-2 -7.82	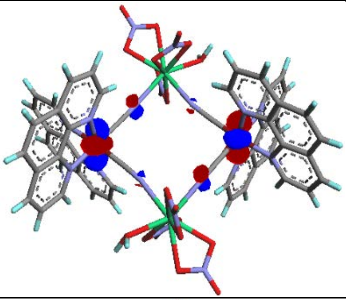	HOMO-3 -7.82	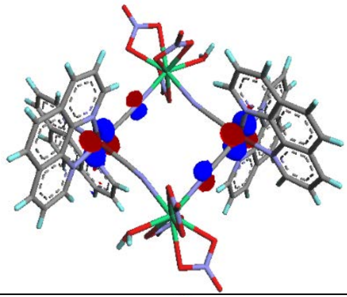
HOMO-4 -7.83	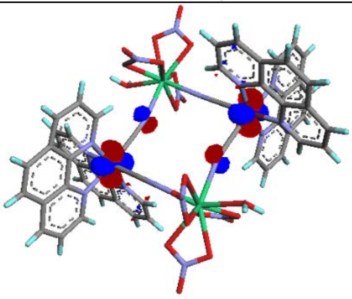	HOMO-5 -7.84	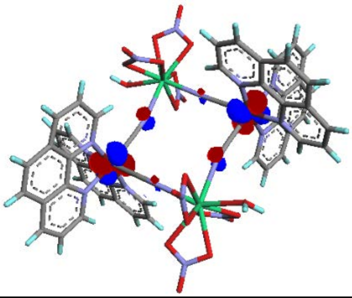

Table S6. DFT calculated molecular orbitals of **2**-Er (isovalue plot 0.04).

LUMO+3 -3.71	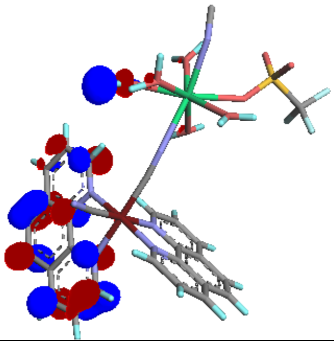	LUMO+4 -3.69	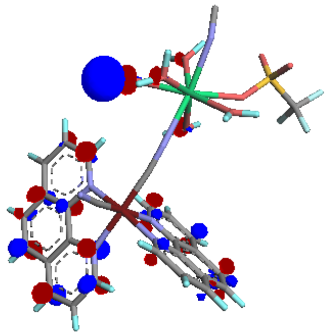
LUMO+1 -4.10	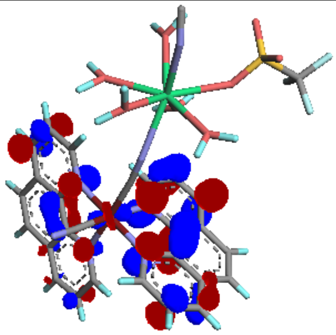	LUMO+2 -3.96	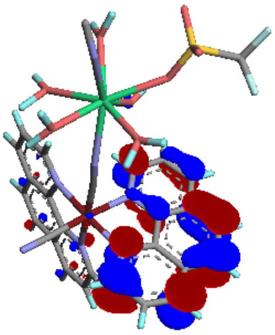
**HOMO -10.06**	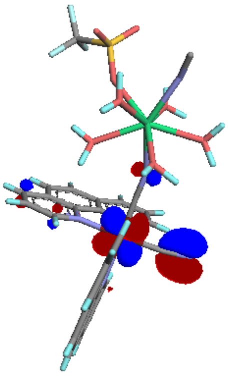	**LUMO -4.20**	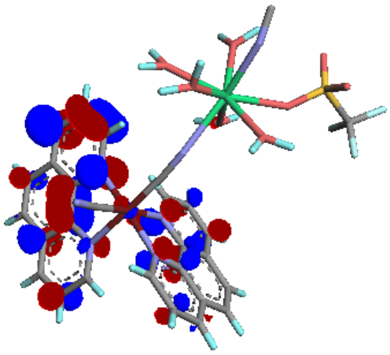
HOMO-1 -10.169	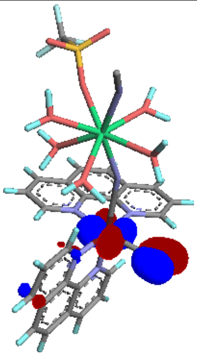	HOMO-2 -10.30	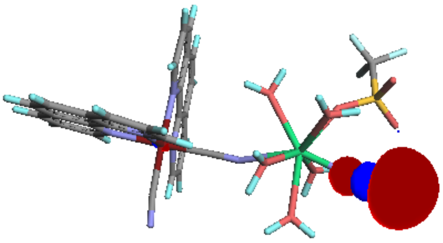
HOMO-3 -10.42	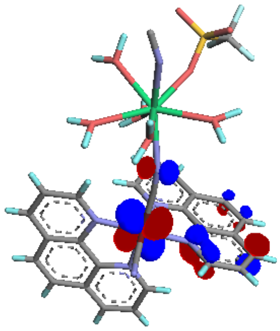	HOMO-4 -10.68	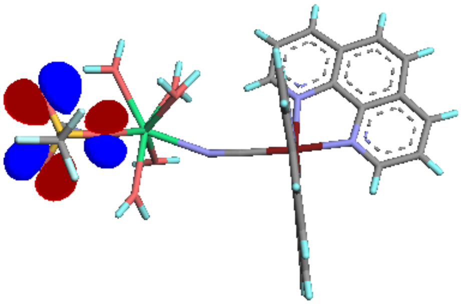
